# Virtual reality-assisted learning: enhancing psychomotor competence in midwifery nursing students

**DOI:** 10.1186/s12912-025-03911-2

**Published:** 2025-10-21

**Authors:** Asmaa Abdallah Ahmed, Mohamed Yehia Ali Mohamed, Heba Ahmed Galal, Nevin Mohamed Mohamed Hasanein, Rania Abouzaid Embaby

**Affiliations:** 1 Department of Maternal and Newborn Health Nursing, Cairo University, Giza, Egypt; 2 College of Nursing, Northern Border University, Arar, Saudi Arabia; 3 Department of Community Health Nursing, Cairo University, Giza, Egypt; 4 Department of Obstetrics and Gynaecological Nursing, Suez Canal University, Ismailia, Egypt

**Keywords:** Midwifery, Nursing students, Psychomotor competence, Virtual reality

## Abstract

**Introduction:**

The integration of virtual reality (VR) in nursing and midwifery teaching has emerged as a promising global trend. Educators have adopted VR to enhance students’ education and ensure safe clinical practice.

**Aim:**

To evaluate the impact of virtual reality-assisted learning on enhancing psychomotor competence among midwifery nursing students.

**Methods:**

A quasi-experimental study was conducted at the Faculty of Nursing, Suez Canal University, specifically in the simulation clinical laboratory and the antenatal outpatient clinic of the university hospital. Stratified random sample of 60 midwifery undergraduate nursing students were divided equally into study and control groups. Data collection occurred between September 2024 and January 2025 using five tools: a self-administered questionnaire, the System Usability Scale, Leopold’s maneuver checklist, an antenatal maternal physical assessment tool, and a student satisfaction questionnaire.

**Results:**

VR-assisted training led to higher post-test knowledge scores compared to traditional methods, though the difference was not statistically significant (*p* = 0.35). There was no statistically significant difference between the study and control groups in psychomotor competence for both Leopold’s maneuver and maternal assessment prior to the intervention (*p* = 0.11 and *p* = 0.07, respectively). However, following the VR intervention, the study group demonstrated significantly higher psychomotor competence in Leopold’s maneuver (43.13 ± 1.46; 42.6 ± 3.82) and maternal physical assessment (36.33 ± 1.81; 37.6 ± 0.67) compared to the control group (36.53 ± 2.29; 32.77 ± 4.28) and (30.13 ± 3.98; 27.3 ± 3.96) in immediate and follow-up posttests respectively (*p* = 0.000). Additionally, 97% of the study group achieved Grade A (superior psychomotor performance) based on system usability with VR, and 63.3% reported being extremely satisfied with the VR learning experience.

**Conclusion:**

The study concluded that midwifery nursing students who received virtual reality-assisted training demonstrated better psychomotor competence, superior performance, and greater satisfaction compared to those trained using traditional simulation methods. However, although the VR group showed higher knowledge scores, the difference was not statistically significant.

**Recommendation:**

It is recommended to incorporate VR-assisted learning into midwifery and maternal health education to enhance students’ psychomotor skills. Ongoing collection of student feedback is essential for continuous improvement of the VR learning experience.

## Introduction

Psychomotor competence, which includes technical skill, motor coordination, and clinical decision-making, is a critical component of effective nursing and midwifery education. Despite being widely regarded as the gold standard, traditional laboratory training and clinical rotations frequently fail to close the gap between theoretical knowledge and clinical preparation in the actual world [1, 2]. Persistent issues include restricted access to clinical sites, ethical constraints, and patient safety concerns further worsen this separation. Alternative teaching strategies that offer students high-fidelity, secure, and replicable possibilities for experiential learning are therefore becoming more and more necessary [3].

Virtual reality-assisted learning, or VRAL, has become a viable teaching technique to help with these issues. Through the use of VRAL, students can immerse themselves in clinical simulations, allowing them to frequently rehearse intricate procedures and interact with high-risk situations. In addition to improving decision-making, confidence, and procedural correctness, this also fosters the growth of crucial non-technical abilities including communication, critical thinking, and situational awareness [4]. VRALs incorporation into healthcare education is not without challenges, though. Widespread implementation is nevertheless hampered by high equipment costs, the requirement for specialized faculty training, greater cognitive load, and challenges integrating VRAL with conventional curricula [5].

The growing popularity of VR around the world highlights how applicable and viable it is becoming in educational settings. By 2023, there was over 65 million VR users, up from 22.5 million in 2017 [5]. Although the entertainment and gaming industries are largely responsible for this rise, it also indicates that VR platforms are becoming more widely available for integration into education. Systematic reviews and meta-analyses in the nursing and midwifery fields have validated the superiority of virtual reality (VR) over traditional approaches in enhancing clinical preparation and procedural knowledge. Virtual reality has proven to be effective in facilitating the acquisition of skills for a variety of procedures, such as intravenous insertion, chemotherapy treatment, and labor management [6].

Empirical studies further substantiate these findings. A quasi-experimental study conducted in Ghana found that VRAL simulations covering birthing scenarios from labor management to placental inspection significantly improved midwifery students’ clinical preparedness, psychomotor competence, confidence, technical ability, and decision-making [7]. Similarly, a pilot usability survey of 43 UK undergraduate midwifery students reported high satisfaction with the VR system (mean SUS = 75.9), noting its effectiveness as a safe and practical method for practicing complex clinical procedures before placements [8].

In order to improve learning outcomes and instructional coherence, the current study integrates Virtual Reality-Assisted Learning (VRAL) into Kolb’s Experiential Learning Theory, building on prior research. Concrete experience, reflective observation, abstract conceptualization, and active experimentation are the four stages of Kolb’s paradigm, which promotes deeper cognitive involvement and works well in practice-based fields like midwifery. This method guarantees that midwifery nursing students ponder, comprehend, and apply abilities in authentic clinical settings in addition to completing tasks digitally. Kolb’s paradigm improves clinical judgment, retention, and the learning experience’s applicability in real-world situations [9].

Unfortunately, VRAL is still not widely used in midwifery, despite its growing use in nursing education. This is especially true when it comes to teaching crucial obstetric procedures like Leopold’s maneuvers and antenatal maternal assessment. Few research have looked at whether these advances in knowledge and student satisfaction translate into long-term performance in clinical assignments, despite the fact that numerous studies find excellent outcomes in these areas. The transferability of VR-acquired competencies to real-world clinical settings is particularly lacking in longitudinal, performance-based research [10, 11].

In order to close that gap, this study assesses the efficacy of a VRAL module that teaches Leopold’s maneuvers, antenatal maternal assessment and is based on Kolb’s experiential learning theory. The study not only assesses short-term learning outcomes but also looks at how well the abilities learned by VRAL are kept and used in actual clinical settings. By using a well-established educational model to the integration of VR in midwifery education, it makes a theoretical contribution. Additionally, it offers empirical evidence of the educational influence of VRAL on clinical competence.

## Theoretical background

Kolb’s Experiential Learning Theory [12] outlines learning as a four-stage cycle: concrete experience, reflective observation, abstract conceptualization, and active experimentation. This model effectively frames how midwifery nursing students acquire psychomotor skills such as Leopold’s Maneuvers and antenatal assessments through VR-Assisted Learning (VRAL) Kolb, D. A. [12].

The Concrete Experience was applied as VRAL Group (Study Group engaged with pre-loaded 3D VR modules demonstrating Leopold’s Maneuvers and antenatal assessments, followed by hands-on practice using high-fidelity manikins. This provided an immersive and authentic learning environment where students physically performed assessments while visualizing internal fetal positions, enhancing their tactile and spatial understanding. The experience was carefully controlled (10-minute intervals) and supervised, ensuring structured exposure and immediate outcome evaluation using psychomotor tools). After the simulation and practical exercises, students embarked on the reflective observation stage, during which they assessed and reflected on their learning experience. During this phase, students were motivated to analyze their performance critically, recognize challenging aspects, and talk about any doubts they had. Research personnel enabled this reflection via verbal discussions, providing constructive input on students’ technique, timing, and clinical precision. The application of the VR Usability Survey (Tool II) encouraged students to think critically about the effectiveness, usability, and educational benefits of the VR training [13].

During the third phase, students started to combine their experiences and reflections with theoretical knowledge. Before the VR sessions, they underwent organized theoretical training via a 30-minute instructor-led orientation, printed study guides, and skill assessment checklists. These resources highlighted the clinical reasoning behind conducting Leopold’s Maneuvers and prenatal evaluations, focusing on how the results influence obstetric choices. In the VR modules, theoretical concepts were integrated into the visual displays, aiding students in mentally linking their actions to the underlying reasons for those actions. Through this process, students moved from mechanical practice to deeper conceptual insight, grasping the importance of fetal positioning, presentation, maternal assessment and engagement in ensuring safe maternal care.

The concluding phase of Kolb’s model, active experimentation, was implemented in the evaluation and follow-up stage of the research. A week following the intervention, students utilized their newly learned skills in a practical environment at the outpatient antenatal clinic of Suez Canal University Hospital. Every participant performed an antenatal evaluation on a pregnant woman (gestational age 28–32 weeks), following the identical order of Leopold’s Maneuvers and physical examination procedures utilized in VRAL. Researchers assessed and analyzed student performance utilizing a validated clinical skills checklist during 10-minute timed sessions. After this real-world application, students filled out the VR Satisfaction Survey, evaluating how effectively the VR experience equipped them for clinical practice [14].

## Research gap and significance of the study

It is becoming more widely acknowledged that virtual reality-assisted learning (VRAL) can improve midwifery students’ psychomotor skills, particularly in light of ongoing difficulties obtaining clinical practice opportunities brought on by changing healthcare environments, ethical considerations, and patient safety concerns [3]. VRAL presents a viable substitute that facilitates more in-depth experiential learning by permitting safe error-making, reflection, and repeated practice [15].

Nevertheless, despite increased interest, not many research have used a systematic pedagogical framework to direct the design and implementation of VRAL in midwifery education, such as Kolb’s Experiential Learning Theory [10]. Furthermore, the majority of current research focuses on short-term knowledge gains or student happiness, with little attention paid to how VR-trained abilities transition into clinical performance. Studies on crucial obstetric techniques, such as Leopold’s maneuvers and antenatal maternal assessment, a basic but sometimes neglected skills during prenatal care, and how students implement this knowledge during practical clinical placements are particularly lacking [16].

So, this study uses Kolb’s experiential learning paradigm to build and assess a VRAL module on Leopold’s maneuvers, antenatal maternal assessment and measures skill transfer to clinical practice in a new way. It provides affordable, scalable training while making theoretical and empirical contributions to midwifery education. It promotes evidence-based curriculum development and equips students with increased competence and confidence by improving clinical experience in a safe and immersive manner [7].

## Aim of the study

The aim of the current study was to evaluate the impact of virtual reality-assisted learning on enhancing psychomotor competence among midwifery nursing students.

### Objectives

1. To assess and compare the baseline and post-training levels of knowledge related to psychomotor competence between students receiving virtual reality (VR) training and those receiving traditional training.
2. To assess the baseline level of psychomotor competence among midwifery nursing students before VR training.
3. To compare post-training psychomotor competence between students trained with VR and those trained using traditional methods.
4. To evaluate the satisfaction of midwifery nursing students with VR-assisted learning as a clinical training approach.

### Operational definition

For the purpose of the current study, the following operational definitions were addressed:

- **Psychomotor competence**: the ability to perform clinical skills with an effective, safe and consistent manner by integrating knowledge into skills to achieve proficiency in specific procedures while adhering ethical rules. Competency was evaluated during antenatal clinical round and using validated assessment checklists.
- **Virtual reality assisted learning:** It is implemented by having students wear VR glasses through mobile phones to watch view pre-downloaded videos 3D that demonstrate each step of procedure. Simultaneously, they practiced these steps on a simulation mannequin in the clinical skills laboratory.”

### Research hypothesis

To achieve the aim of this study, the following research hypotheses were formulated:

#### H1

Midwifery nursing students who undergo virtual reality (VR) training will exhibit significantly higher competency levels in specific psychomotor skills compared to students receiving traditional clinical training.

#### H2

Midwifery nursing students who undergo virtual reality (VR) training will report significantly greater satisfaction with their training compared to those who receive traditional training.

### Research design

In the current study, a quasi-experimental study approach was employed. A type of research used to examine the impact of an intervention is a quasi-experimental design. Two groups are often included in this design: one receives a novel intervention (like virtual reality-assisted learning) and the other receives the conventional approach (like traditional instruction). Prior to the intervention (pre-test) and following the intervention (post-test), researchers assess each group’s performance or skill level. They can determine whether the new approach resulted in a higher improvement by comparing the outcomes [17].

### Setting

The current study was carried out at the obstetrics and gynecology laboratory skills at Faculty of Nursing, Suez Canal University and antenatal outpatient clinic affiliated to Suez Canal University hospital to apply the procedures acquired on real pregnant mothers at hospital. The obstetrics and gynecology laboratory is located on the third floor of the educational building. It contains six beds with obstetrics and gynecology mannequins for practice clinical procedure. Each bed accommodates 6 students during the lesson. Antenatal outpatient clinic contains two rooms. The first room has a desk and chairs for taking history. The other room has two parts: the first is an examination bed and the other is a bed with an ultrasound machine and capacity of room contains 3 students.

### Study sample

The study sample was selected based on the following inclusion criteria: Third level student, either male or female student and didn’t use VR technologies in applying Leopold’s maneuver or maternal physical assessment procedure before. pregnant women with GA 28-37wks. While students with previous knowledge and skills about clinical procedures and did not register midwifery curriculum, pregnant women with GA less than 28 wks.; twins pregnancy; Visual acuity uncorrectable; Active vestibular disorders; Missed scheduled training session were excluded.

The sample size was determined using G*Power version 3.1.9.7 for a one-tailed t-test comparing the means of two independent groups. An effect size (d) of 0.79 was assumed, based on prior findings by Oh and Kim [18] suggesting a moderate-to-large expected difference. The analysis utilized a significance level (α) of 0.05 and a power (1–β) of 0.90, with an assumption of equal allocation ratio between groups (N2/N1 = 1). The estimated total sample size was 60 participants, comprising 30 individuals in each group.

### Sampling technique

A stratified random sampling technique was utilized to guarantee sufficient representation from all students participating in midwifery clinical training. The total population (*N* = 120) was divided into four groups based on specialty: antenatal, high-risk pregnancy, childbirth, and postpartum. A sample of 60 students was drawn from the antenatal clinical training group through proportional stratified random sampling, with 15 students randomly selected from each of the four training lists.

After selection, students were randomly divided into two equal groups: a study group (*n* = 30) and a control group (*n* = 30). This was achieved through simple random assignments, such as computer-generated random numbers, to reduce selection bias and maintain comparable baseline characteristics across groups.

The study group was provided with theoretical content regarding antenatal maternal physical assessment and Leopold’s maneuvers, subsequently engaging in skills training through Virtual Reality (VR)-assisted learning. The control group was provided with identical theoretical instruction, followed by a conventional demonstration of the clinical procedure by an instructor, and subsequently re-demonstrated the skill under supervision as part of standard clinical learning. This design-maintained uniformity in theoretical content while isolating the impact of the instructional method on the acquisition of clinical skills.

### Tools for data collection

Five main tools were utilized to acquire data for this study:

**Tool 1. Self-administered questionnaire:** It involves two parts:

*Part1. Personal profile*: It contained data on demographic characteristics (age, gender, place of residence, phone number and previous experiences related to virtual reality.

*Part2. Knowledge assessment related to psychomotor competence:*

The researcher developed this tool following an extensive review of recent literature to evaluate midwifery students’ knowledge related to Leopold’s maneuvers and associated maternal-fetal assessment concepts. The tool specifically assesses:

- The purpose of each grip in Leopold’s maneuvers.
- Definitions of key terms (e.g., *fetal lie, attitude, position, presentation*).
- Maternal physical assessment markers (e.g., *Linea nigra, striae gravidarum*).

Tool Structure: 12 multiple-choice questions (MCQs), each with four alternative options.

Scoring: 1 point per correct answer (total score range: 0–12).Cut-off score: ≤6 = Unsatisfactory knowledge level. & > 6 = Satisfactory knowledge level (based on [19]). It is used for pre-test and post-test evaluation. Cronbach’s Alpha = 0.78 (indicating good internal consistency for the 12-item scale).

### Tool II. System usability scale (SUS)

This is standardized tool adopted from [8]. It is commonly used to evaluate the usability of a technology, including Virtual Reality (VR).The SUS consists of 10 items, with items 1, 3, 5, 7, and 9 worded positively (favoring VR), and items 2, 4, 6, 8, and 10 worded negatively (not favoring VR).

#### Scoring system

Items are scored on a 5-point Likert scale ranging from “strongly disagree” (score of 1) to “strongly agree” (score of 5). Negatively worded items are reverse coded; meaning 1 point is subtracted from the original score for these items. These result of each item being scored from 0 to 4. The scores for all 10 items are added and then multiplied by 2.5, producing an overall score ranging from 0 to 100.

#### Interpretation of scores

Higher scores indicate greater usability; SUS scores are converted to percentiles (0–100) and graded (F to A+); Grade a represents “superior performance,” grade C indicates “average performance,” and grade F indicates “failing performance.”

Reliability for this tool by the Cronbach’s α of 0.77 suggests high reliability for the SUS [8].

### Tool III. Leopold’s maneuver checklist

It was developed by a committee of obstetrics and gynecology professors with high content validity (CVI = 95%). It was designed for clinical evaluation of midwifery students’ procedural skills. Reliable (Cronbach’s Alpha = 0.83), confirming strong internal consistency. Total Items: 22, categorized into three phases:

1. Preparation (Items 1–6) – Pre-procedure setup and readiness.
2. Procedure Execution (Items 7–18) – Core steps of the skill (e.g., Leopold’s maneuvers).
3. Post-Procedure (Items 19–22) – Documentation, patient care, and cleanup.

Scoring System Per-Item Scoring: 0 = Incorrect/Not done; 1 = Correct but incomplete; 2 = Correct and fully performed in proper sequence. Total Score Range: 0–44, interpreted as:

0–14 = Unsatisfactory performance; 15–29 = Competent performance& 30–44 = Proficient performance. The reliability for this tool was calculated by Cronbach’s Alpha; it was 0.83 for 44 items.

### Tool IV. Antenatal maternal physical assessment checklist

This validated 19-item checklist was developed by an obstetrics/gynecology expert committee (CVI = 95%) for assessing midwifery nursing students’ clinical skills. The tool evaluates three procedural phases: preparation (items 1–2), main procedure execution (items 3–15), and post-procedure actions (items 16–19). Each item is scored 0 (incorrect/not done), 1 (incomplete), or 2 (correct and complete), yielding a total score of 0–38. Performance levels are categorized as: unsatisfactory (0–19), competent (20–28), and proficient (29–38). The tool demonstrates good reliability (Cronbach’s α = 0.77), making it suitable for objective skills assessment in clinical education and research contexts.

### Tool V: Student’s satisfaction questionnaire

A validated satisfaction tool, adapted from Saab et al. [8] was used to assess students’ experiences with virtual reality (VR) training. The tool included two items measuring satisfaction with learning nursing/midwifery clinical scenarios through VR and overall experience with the study, both rated on a 5-point Likert scale (1 = Extremely Dissatisfied to 5 = Extremely Satisfied). Total scores ranged from 2 to 10, with higher scores indicating greater satisfaction. The tool demonstrated good internal consistency, with a Cronbach’s alpha of 0.88. An additional open-ended question asked students to identify the most enjoyable procedure learned through VR-assisted training.

**Table:** 

Tool	Purpose	Items	Validity	Reliability (α)	Scoring
Knowledge Assessment	Theoretical mastery	12 MCQ	CVI = 0.95*	0.78	0–12 (Cutoff: > 6)
Leopolds maneuver Checklist	Procedural skills	22 steps	CVI = 0.95*	0.83	0–44 (Competent: ≥15)
Maternal physical assessment Checklist	Procedural skills	19		0.77	0–38 Proficient ≥29
VR Satisfaction Scale	User acceptability	2 Likert	–	0.88	2–10 (Higher = better)

*Content Validity Index from expert panel in the field of maternity nursing

### Ethical consideration

The current study received ethical approval from the Research Ethics Committee of the Faculty of Nursing, Suez Canal University in July 2024, including approval of the study 277/7/2024

The study was conducted in accordance with the Declaration of Helsinki. The researcher clearly explained the study’s purpose, nature, and significance to the students. Informed consent was obtained from students who voluntarily agreed to take part, with assurance that their involvement posed no risk or harm emphasized no penalty for withdrawal. Students were also informed of their right to withdraw from study at any time without any negative consequences. In addition, all data were coded using ID numbers and securely stored by the researcher to maintain confidentiality.

### Procedure of data collection

Following approval from the Research Ethics Committee at the Faculty of Nursing, Suez Canal University, formal authorization was obtained through official correspondence with the Dean and Vice Dean for Education and Student Affairs. Data collection occurred between September 2024 and January 2025.The study implementation followed a structured four-phase protocol: Preparation Phase: Instrument validation and pilot testing; Baseline Assessment Phase: Initial data collection through structured interviews; Intervention Phase: Controlled implementation of the experimental protocol& Evaluation Phase: Evaluation and follow-up measurements.

### Preparation phase

The study instruments were systematically developed through an extensive literature review and subsequently validated via expert review: The tools underwent rigorous evaluation by a panel of three maternity nursing specialists to ensure content validity and clinical relevance. In addition, Researcher Training: All investigators completed a standardized VR competency program, including Didactic orientation on VR-assisted learning principles, Hands-on simulation training & Proficiency assessments for equipment operation and protocol adherence and finally all instruments were pretested to establish reliability before full implementation

### Interview and baseline assessment phase

The research team implemented a structured recruitment and data collection protocol: Participant Engagement: Researchers formally introduced themselves to all eligible midwifery nursing students, after that, conducted in-person informational sessions detailing: Study aims and scientific significance, Potential contributions to educational practice, Voluntary nature of participation, Assurance of no academic consequences, allowed 24-hour consideration period before enrollment & addressed all participant questions through Q&A sessions. Finally, conducted face-to-face interviews using validated instruments: Tool I, Tool II:, Tool III & Tool IV. Maintained consistent administration conditions: Private clinical skills lab setting, 45–60-minute sessions.

### Implementation phase

#### Standardized theoretical training (both groups)

All participants received: Identical didactic materials covering for both psychomotor skill competence as Leopold’s maneuvers theory and antenatal physical assessment protocols, Printed checklists and learning guides & 30-minute instructor-led orientation session.

#### VR-assisted learning (study group)

VR Setup & Orientation Conducted in preserved time in which it was free of clinical lab training and Structured 10-minute equipment briefing Hygiene protocols for VR headset use & Safety precautions Simulation Training Protocol

1. Immersive Learning Component:
  Viewed pre-loaded VR training modules (10 minutes)
  Content included:3D video demonstrations of Leopold’s maneuvers & Guided antenatal assessment procedures
2. Hands-on Practice:

Simultaneous practice on high-fidelity manikins & Researcher-supervised sessions ensuring Procedural accuracy; Time management (strict 10-minute intervals) & Immediate Outcome Assessment Psychomotor Evaluation (Tools III & IV) which performed on manikins under observation and Knowledge Post-test (Tool I) & VR Usability Survey (Tool II)

**Fig. 1:**
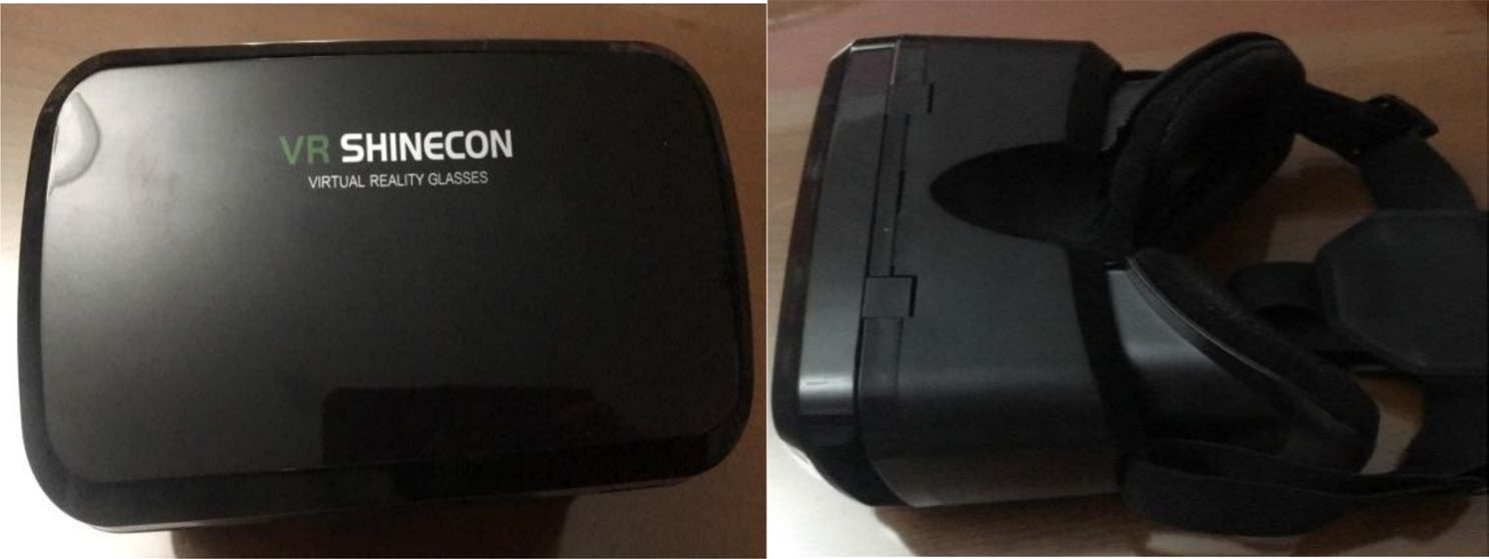
Shinecon VR

This module is commercially available products that are adapted for a study. This could be a pre-existing VR game or a training simulation. Shinecon VR is a brand of virtual reality headsets designed primarily for mobile VR experiences. The headsets are built to work with smartphones, allowing users to enjoy immersive 3D videos, and virtual environments at a relatively low cost (Fig. 1).

### Control group (traditional training) implementation

**Standardized Demonstration Phase:** Researchers performed comprehensive demonstrations on high-fidelity simulation mannequins: Leopold’s maneuvers (all four grips) in addition, antenatal physical assessment procedure. Each procedure was demonstrated twice with accompanied by verbal explanations of key anatomical landmarks with considered timed to match VR group’s total exposure (30 minutes). For practice Session: Participants conducted supervised re-demonstrations featuring as well as immediate corrective feedback, Checklist-guided performance and identical manikin models as VR group. Duration of practice was controlled (10 minutes/procedure). After that, psychomotor competence Assessment (Tools III & IV) and Knowledge Post-test (Tool I): 12-item MCQ administered electronically Time-limited to 15 minutes

### Evaluation and follow up phase

**Fig. 2:**
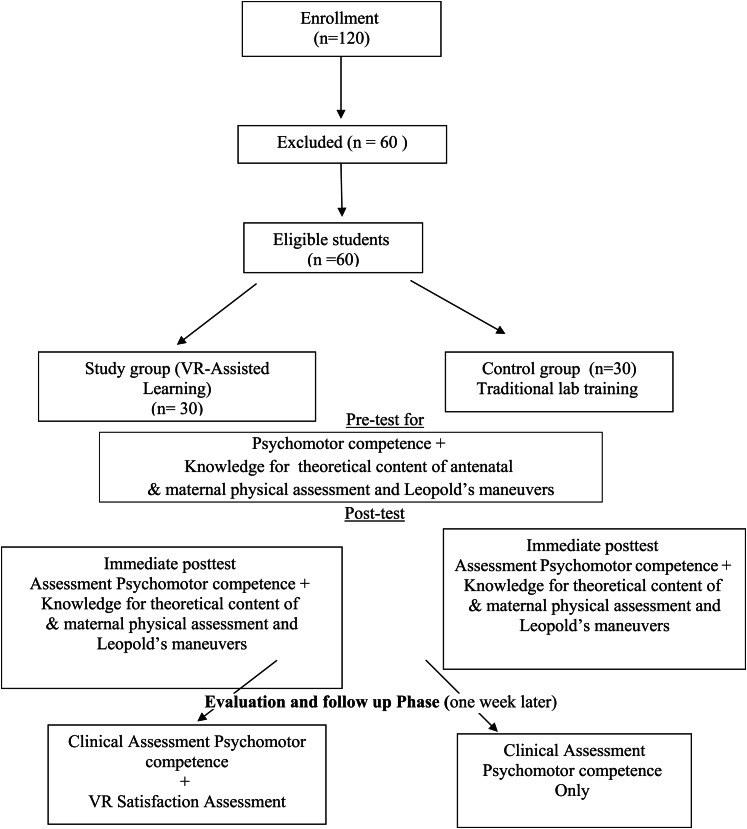
CONSORT

During this phase, Clinical Competency Assessment was conducted one week later post-intervention at Outpatient antenatal outpatient clinic affiliated to Suez Canal University hospital. During this phase pregnant women (GA 28–32 weeks) was consented then researchers evaluated both of study and control midwifery nursing students using validated checklist within 10-minute observation. VR Satisfaction Assessment (study group only) was also assessed using tool V in private post-evaluation (Fig. 2).

### Statistical analysis

All analyses were conducted using SPSS Statistics version 26 (IBM Corp.). Descriptive statistics for categorical variables, such as demographic characteristics, were presented as frequency distributions (%). Virtual reality (VR) experience profiles were analyzed using cross-tabulations with the Chi-square (χ^2^) test.

Psychomotor competency scores were reported as mean ± standard deviation (SD). The normality of continuous variables was assessed using the Shapiro-Wilk test, with p-values greater than 0.05 indicating an approximately normal distribution. Despite the relatively small sample size, which can limit the power of normality tests, the data did not show significant deviations from normality. Therefore, parametric tests were deemed appropriate for analysis.

Accordingly, within-group changes from pre-test to post-test were evaluated using paired t-tests, and between-group comparisons were conducted using independent samples t-tests for continuous variables. For categorical variables, the Chi-square test was used. The alpha level was set at 0.05 (two-tailed), with p-values less than 0.05 considered statistically significant and p-values less than 0.01 considered highly significant. All statistical tests were performed with a power of 95%.

## Results

Table 1 presents baseline demographic characteristics of the study and control groups. The two groups were well matched, with no statistically significant differences observed in age, gender, or prior VR experience (all *p* > 0.05). The mean age was 20.9 ± 0.48 in the study group and 20.6 ± 0.55 in the control group (*p* = 0.06). Both groups were predominantly female, with no significant difference in gender distribution (*p* = 0.27). Additionally, 86.7% of the study group and 60% of the control group reported no prior VR experience (*p* = 0.13).

**Table 1:** Demographic characteristics of the study samples (*N* = 60, 30 each)

Characteristics	Study group		Control group		X2	P
	N	%	N	%		
**Age**					4.1	0.12
20 year	5	16.7	12	40.0		
21 year	23	76.7	17	56.7		
22 year	2	6.7	1	3.3		
Mean ± SD	20.9 ± 0.48		20.6 ± 0.55		t = 1.9	0.06
**Gender**					1.1	0.27
Male	3	10.0	6	20.0		
Female	27	90.0	24	80.0		
**Previous experience with virtual reality**						
None	26	86.7	18	60.0	5.5	0.13
used once	1	3.3	4	13.3		
used several times	2	6.7	5	16.7		
used a lot	1	3.3	3	10.0		

No significant at *p* > 0.05. *Significant at p ‹ 0.05. **highly significant at p ‹ 0.01

Power = 95% Alpha level = 0.05

**Fig. 3:**
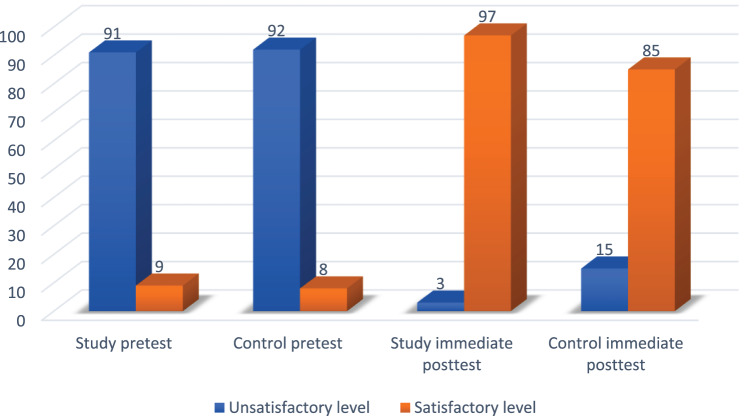
Knowledge of both group’s pretest and immediate post-test through the study phases

This figure highlights the distribution of theoretical knowledge related to maternal physical assessment and Leopold’s maneuver across the study and control groups. At the pre-test phase, 91% of the study group and 92% of the control group demonstrated unsatisfactory knowledge, indicating limited understanding of the theoretical concepts. Following the intervention, 97% of the study group reached a satisfactory knowledge level compared to 85% in the control group. Although this suggests an improvement in theoretical understanding within the study group, the difference between groups was not statistically significant (*p* = 0.35), as shown in Fig. 3. It is important to note that this measure reflects only theoretical knowledge, distinct from the psychomotor competence scores, which are reported separately below and assess practical skill performance.

Table 2 shows the psychomotor competence of Leopold’s maneuver at two time points. The study group achieved significantly higher mean scores (43.13 ± 1.46 and 42.6 ± 3.82) compared to the control group (36.53 ± 2.29 and 32.77 ± 4.28) in the immediate post-test and follow-up post-test, respectively, with a statistically significant difference between groups (*p* = 0.000). The effect sizes were large (3.6 immediate post-test, 2.5 follow-up), indicating a substantial and sustained improvement in the study group. Similarly, for maternal physical assessment, the study group attained higher mean scores (36.33 ± 1.81 and 37.6 ± 0.67) than the control group (30.13 ± 3.98 and 27.3 ± 3.96) at the immediate post-test and follow-up post-test, respectively, also with statistically significant differences (*p* = 0.000). Effect sizes ranged from moderate to very large (2.2 immediate post-test, 4.4 follow-up), demonstrating meaningful gains and retention of skills in the study group over time.

**Table 2:** Scores of both groups at study phases in relation to psychomotor competence

Psychomotor Competence	Pre – test				Immediate Post - test				Follow up post – test			
	Study group		Control group		Study group		Control group		Study group		Control group	
	M	SD	M	SD	M	SD	M	SD	M	SD	M	SD
Leopold’s maneuver	11.77	3.20	3.27	2.73	43.13	1.46	36.53	2.29	42.6	3.82	32.77	4.28
Effect size	1.5				3.6				2.5			
	t = 1.62 *P* = 0.11 CI = 6.96 to 10.04				t = 5.309 p = 0.000* CI = 5.61 to 7.59				t = 9.385 *P* = 0.000* CI = 7.73 to 11.93			
Maternal physical assessment	9.03	2.18	3.97	2.94	36.33	1.81	30.13	3.98	37.6	0.67	27.3	3.96
Effect size	0.9				2.2				4.4			
	t = 0.80 *P* = 0.07* CI = 3.72 to 6.40				t = 6.66 *P* = 0.000* CI = 4.60 to 7.80				t = 13.7 *P* = 0.000* CI = 8.83 to 11.77			

No significant at *p* > 0.05. *Significant at p ‹ 0.05. **highly significant at p ‹ 0.01

Power = 95% Alpha level = 0.05

**Fig. 4:**
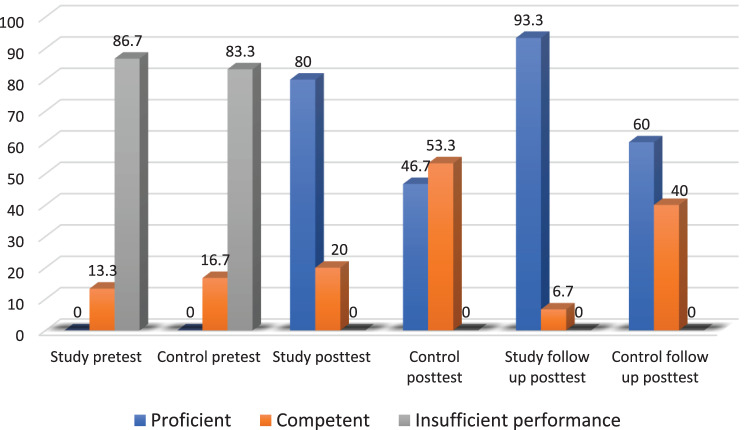
Performance levels regarding specific psychomotor competence (Leopold’s maneuver) through the study phases

Figure 4 illustrates the variations in performance levels for Leopold’s Maneuver in both the study and control groups during the pre-test, immediate post-test, and follow-up periods. At baseline, most students in both groups showed insufficient performance (86.7% in the study group compared to 83.3% in the control group), with no statistically significant difference (*p* = 0.93). After the intervention, 80% of the group trained with virtual reality reached proficient performance, in contrast to 46.7% in the control group, demonstrating a statistically significant difference (*p* = 0.001). At the one-week follow-up, the study group’s proficiency rose to 93.3%, whereas the control group achieved 60%, showing a markedly significant difference (*p* = 0.001).

**Fig. 5:**
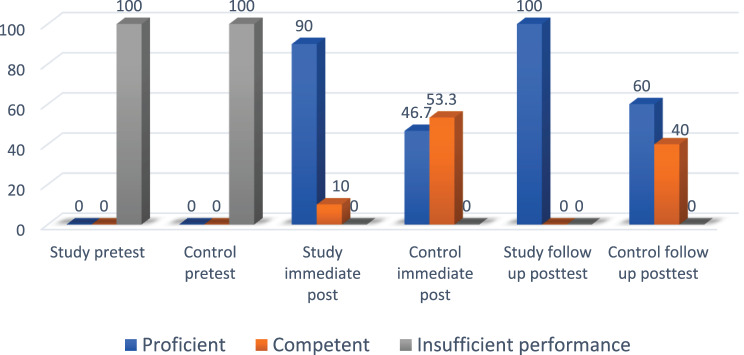
Performance levels regarding specific psychomotor competence (maternal physical assessment) through the study phases

Figure 5 illustrates the performance distribution of maternal physical assessment for the study and control groups during the pre-test, immediate post-test and follow-up phases. At first, all students in both groups exhibited inadequate performance, showing no notable difference between them (*p* = 1.0). Right after the intervention, 90% of the study group, trained with virtual reality, reached proficient performance, whereas only 46.7% of the control group, who underwent traditional manikin simulation, achieved the same, demonstrating a highly significant difference (*p* = 0.001). At the one-week follow-up, every student in the study group (100%) sustained proficiency, whereas 60% of the control group attained this level, again showing a highly significant difference between groups (*p* = 0.001)

Table 3 The System Usability Scale (SUS) results indicated that students had a highly positive experience with Virtual Reality-Assisted Learning (VRAL). Nearly all participants (96.7%) expressed a strong desire for frequent use, reflecting high engagement and interest. The vast majority (86.7%) disagreed that the system was overly complex, and all students (100%) found it easy to use and quick to learn. Most students (93.3%) felt they did not require assistance to operate the VR system, and 80% believed its functions were well-integrated. Additionally, 93.3% disagreed that the system was inconsistent, and 86.7% felt it was not cumbersome. Confidence levels were also high, with 90% reporting strong confidence in using the VRAL. However, only 40% disagreed that prior learning was needed before starting, suggesting that some students still perceived a slight learning curve.

**Table 3:** System usability scale of study group (*n* = 30)

Items	Strongly disagree		Somewhat Disagree		Neutral		Somewhat agree		Strongly agree	
	No.	%	No.	%	No.	%	No.	%	No.	%
I think I would like to use VR simulation frequently	1	3.3	0	0.0	0	0.0	0	0.0	29	96.7
I found VR simulation unnecessarily complex	26	86.7	4	13.3	0	0.0	0	0.0	0	0.0
I thought VR simulation was easy to use	0	0.0	0	0.0	0	0.0	0	0.0	30	100.0
I think that I would need assistance to be able to use VR simulation	28	93.3	2	6.7	0	0.0	0	0.0	0	0.0
I found the various functions in VR simulation were well Integrated	0	0.0	0	0.0	0	0.0	6	20.0	24	80.0
I thought there was too much inconsistency in VR simulation	28	93.3	1	3.3	1	3.3	0	0.0	0	0.0
I would imagine that most people would learn to use VR simulation very Quickly	0	0.0	0	0.0	0	0.0	0	0.0	30	100.0
I found VR simulation very cumbersome/awkward to use	26	86.7	0	0.0	3	10.0	0	0.0	1	3.3
I felt very confident using VR simulation	0	0.0	0	0.0	0	0.0	3	10.0	27	90.0
I needed to learn a lot of things before I could get going with VR simulation	0	0.0	12	40.0	11	36.7	6	20.0	1	3.3

No significant at *p* > 0.05. *Significant at p ‹ 0.05. ** Highly significant at p ‹ 0.01

**Fig. 6:**
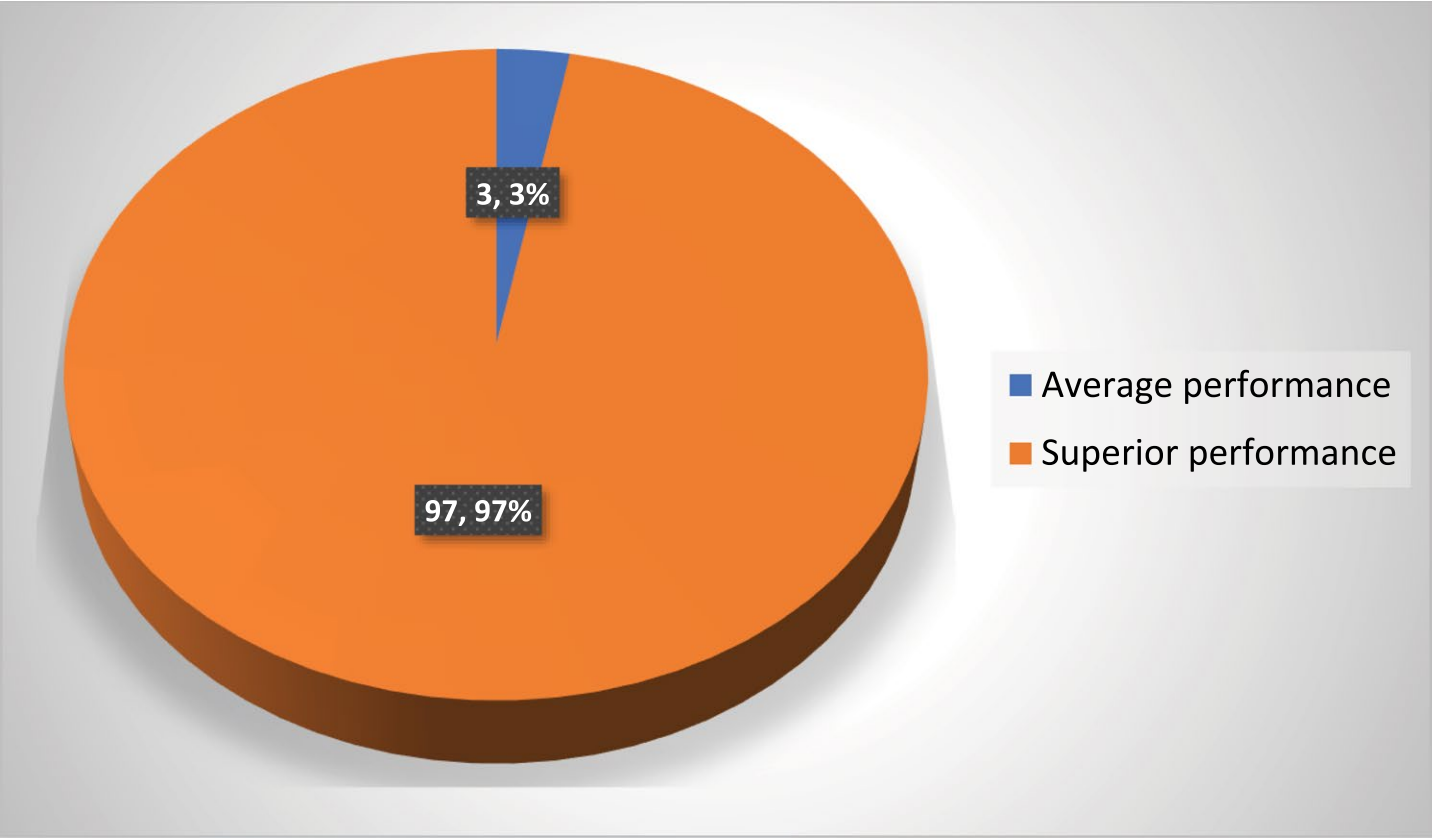
Total percentage of system usability scale of the study group VR assisted learning among the study group (*n* = 30)

Figure 6 illustrates percentage of system usability in VR group. It shows that the majority of the study participants (97%) demonstrated grade A (superior performance), while only 3% exhibited grade C (average performance) after using virtual reality assisted learning using glasses to acquire psychomotor skills for abdominal maneuvers and maternal assessments.

Table 4 shows that, the distribution of satisfaction of VR group towards VR assisted learning, that the highest frequency of participants (63.3%) reported being “Extremely satisfied” with using the VR assisted learning for about nursing/midwifery clinical scenarios. While a smaller proportion (20%) remained neutral, and only 16.7% were “Somewhat satisfied.” In addition the majority of participants (66.7%) were “Extremely satisfied” with their overall experience in the study and 23.3% were somewhat satisfied this reflects a highly positive reception of the study as a whole.

**Table 4:** Satisfaction levels of the study group towards VR simulation

Satisfaction levels	Extremely dissatisfied		Somewhat dissatisfied		Neutral		Somewhat satisfied		Extremely satisfied	
	No.	%	No.	%	No.	%	No.	%	No.	%
Using the virtual reality (VR) simulation to learn about nursing/midwifery clinical scenarios	0	0.0	0	0.0	6	20%	5	16.7%	19	63.3%
My overall experience of participating in this study	0	0.0	0	0.0	3	10%	7	23.3%	20	66.7%

**Fig. 7:**
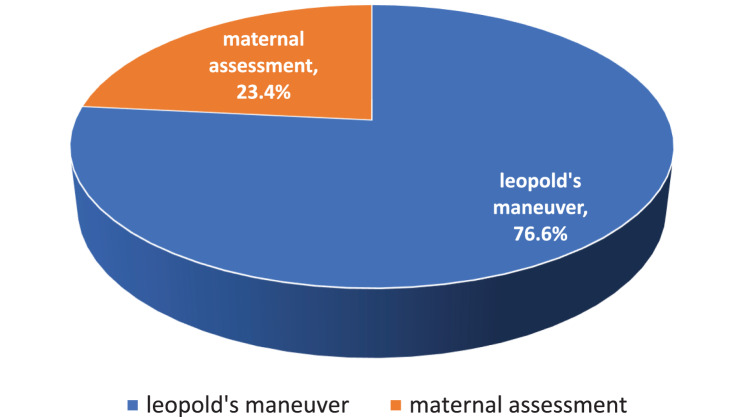
Percentage of most enjoyable elements of VR simulation in psychomotor competence among the study group (*n* = 30)

Figure 7 shows that, three quarter of midwifery nursing students (76.6%) enjoyed performing Leopold’s maneuver with virtual reality assisted learning while, 23.4% enjoyed performing maternal physical assessment with virtual reality.

## Discussion

Virtual reality enables nursing students to fully engage with the virtual environment, which offers a more realistic teaching approach than traditional methods. This approach enhances the psychomotor skills of students, provides constructive learning with prompt feedback, and increases their confidence without posing a risk to pregnant women [20].

Hence, several training methods, including video based virtual reality simulation have been proposed in recent years to cope with the current circumstances [21]. VR is intended to replace mannequin-based simulation training, as it has a positive impact on knowledge of nursing students, self-efficacy, communication abilities, and clinical performance [22]. So, the purpose of this study is to evaluate the effect of integrating Virtual Reality-Assisted Learning on enhancing psychomotor competence among midwifery nursing students.

**Concerning demographic characteristics** of the studied groups, the current study revealed no statistical significant difference between the study and control group that increase consistency of the results. This result was similar to [23] demonstrated that the experimental and control groups exhibited no significant differences in terms of socio-demographic characteristics, which suggests homogeneity.

**Regarding to prior virtual reality (VR) experience**, the current study revealed that the majority of studied midwifery nursing students have previous experience through virtual reality eye glasses. This result may be attributed to virtual reality is anew emerging technology so, most nursing student have previous experience of using VR eye glasses. This result was in agreement with Bai & Manomozhi [24], who found that the majority of studied students have previous experience through virtual reality eye glasses. In the researcher point of view, this result may be due to the fact that this is the generation of artificial intelligence.

**As regard to midwifery nursing students’ knowledge assessment related to Leopold’s maneuver**, throughout the study phases, the result of this study revealed that the majority of the studied students were having low level of knowledge in study and control group regarding selected procedures pre- test stage. While immediately post - test of the training program, it was found that the majority of the studied students have high level of knowledge according to students’ knowledge assessment to Leopold’s maneuver. Results from the current investigation were in agreement with [25] showed the difference in maternity nurses’ CPR knowledge between the pre-, post-, and follow-up evaluations. While she was expecting, the nurse’s knowledge of cardiopulmonary resuscitation significantly increased (*p* = 0.000 for all items). The results of this study were also consistent with [26] showed that there was a highly significant difference (*p* < 0.001) in the mean scores of knowledges about cardiac arrest during pregnancy between the phases before and after the intervention.

**Regarding midwifery nursing student’s performance related to Leopold’s maneuver and antenatal maternal physical assessment**, students in both groups demonstrated insufficient performance in the Leopold’s maneuver procedure and the maternal assessment procedure at the pre – test (86.7% & 83.3%). However, immediately after the posttest, the majority study group (80%) and less than half of the control group (46.7%) had satisfactory level of practice post two weeks of implementation of clinical training program. The study group, who practiced the procedure using virtual reality glasses, achieved proficient performance in conducting Leopold’s maneuver on pregnant women at the antenatal clinic during the follow-up. In contrast with the control group that used traditional manikin simulation in the laboratory. Even though the students take their courses from the clinical instructor; by traditional method through a demonstration and perform re-demonstration technique of procedure does not meet the learning needs of students and also do not provide the permanence and consistency. There was significant difference in student’s performance related to Leopold’s maneuver and antenatal maternal physical assessment in both groups.

Although there was no statistically significant difference in knowledge scores between the study and control groups immediately post-intervention (*p* = 0.35), a significant improvement was observed in psychomotor performance among the VR-trained group, particularly in Leopold’s maneuver and maternal physical assessment. This suggests that VR-based training may have a stronger impact on practical skill acquisition than on theoretical knowledge. This notion aligns with systematic evidence indicating that while VR training enhances clinical skill performance significantly (effect size g = 0.682), its effect on knowledge is smaller (effect size ~ 0.237) [27].

This improvement may be related to the effect of using new clinical training method in nursing student training including video based virtual reality which helped to attract student attention and concentration more than traditional method throughout re-demonstration technique. Moreover, it allowed for repetition of the clinical procedures without feeling of nursing students from being overwhelmed and not having lack interest in boring teaching and learning

This result matched with [23] who revealed that on assessments of teaching satisfaction (*p* = 0.115) and cardiopulmonary resuscitation performance (*p* = 0.451 for chest compressions and *p* = 0.378 for airway management), they did not find a statistically significant difference between the experimental and control groups of nursing students. Immediate and one month after the intervention was implemented, the experimental group demonstrated significantly better cardiopulmonary resuscitation performance, including chest compressions (*p* < 0.001), airway management (*p* < 0.001), and teaching satisfaction (*p* < 0.001), in comparison to the control group. Moreover [28], The discovery was made that virtual reality (VR) instruction could impart cardiopulmonary resuscitation (CPR) skills in an engaging manner, without a loss of proficiency, in comparison to more conventional approaches. As well as, Moreover; Lee [22] described that VR for IV injection showed significantly higher clinical performance compared to students who received training through IV arm simulator. The result matched with [29], the authors show improvement in students’ skills when using simulations. But result was not in agreement with Chao et al. [30], who clarified that there were no significant differences in nursing students skill between nursing students who trained through 3D video based virtual reality simulation and students who trained through traditional method on simulator.

While the current study demonstrated a significant improvement in midwifery students’ performance using virtual reality (VR) in mastering Leopold’s maneuver and antenatal maternal physical assessment, recent literature provides a more cautious interpretation of VRs effectiveness in procedural skill acquisition. For example, a meta-analysis by Kim et al. [31] assessed the impact of virtual simulations versus traditional simulation methods (e.g., manikins and real patients) in medical and nursing education. The results indicated no significant difference in improving procedural skills across the two methods (Standardized Mean Difference = −0.12; 95% CI −0.47 to 0.23). Interestingly, in subgroup analysis, nursing participants actually showed lower gains with virtual simulations compared to traditional methods (SMD = −0.55; 95% CI −1.07 to −0.03; *p* = 0.04). These findings suggest that the added value of VR may lie more in increasing learner motivation, engagement, or confidence rather than enhancing clinical performance per se. Moreover, the novelty of VR could introduce a Hawthorne effect, where learners temporarily perform better due to excitement and increased attention, rather than due to the instructional superiority of the medium itself. Therefore, while VR can be a useful educational tool, its use should be integrated thoughtfully alongside other proven methods to ensure sustainable learning outcomes, especially in high-stakes clinical skills like Leopold’s maneuver.

All of these findings illustrate the significant advantages and benefits of utilizing virtual reality technologies to enhance and enhance the performance of midwifery nursing students. This is a result of the technology’s encouraging features and the ability to apply skills in a pleasurable manner without the dread of making an error. From the perspective of the researcher, using of VR is imperative to help midwifery nursing students get closer to the real environment. Thus, VR is presumed to provide positive benefits in improving clinical skills.

While study highlights the superior performance of the VR-enabled group in Leopold’s maneuver, it’s essential to consider the broader implications of adopting this technology in diverse learning environments—particularly regarding accessibility, scalability, and cost. Implementing VR in rural schools or underfunded settings poses significant challenges. Additionally, the high initial costs are a barrier: VR hardware and software development may cost tens of thousands of dollars, placing it out of reach for many institutions. Even when devices are available, supporting large class sizes is difficult due to hardware limitations and bandwidth constraints—for instance, VR streaming demands sustained high-speed internet, which many schools, especially in underserved areas, lack beyond infrastructure, many schools struggle with limited technical support and the need for staff training to manage and maintain VR platforms effectively. Though efforts such as public–private partnerships, subsidies, and pooled purchasing models have been proposed to ease these challenges, their successful implementation requires strategic planning, policy support, and long‑term commitment. [32] From point of researcher, while VR shows promising results, embedding it sustainably in varied educational settings—like rural schools or large classrooms—demands addressing these structural, financial, and technical barriers

**Concerning midwifery nursing students satisfaction towards VR simulation,** the majority of the participants students was reported being “Extremely satisfied” with using the VR simulation for learning about nursing midwifery clinical scenarios, more than three quarters of midwifery students enjoyed performing Leopold’s maneuver with virtual reality. While, about one quarter of enjoyed performing maternal assessment with virtual reality. This reflects a highly positive reception of the study as a whole.

This result may related to nursing students were easily distracted during traditional method. Also, Knowledge acquired through traditional methods is more likely to be lost, but because VR-based training encourages interaction and involvement with the real world, it may help students retain long-term memory and form deeper impressions. This result agreed with [23] showed that the levels of satisfaction among nursing students before the intervention, there was no discernible difference between the groups (*p* = 0.115). On the other hand, there were noticeable differences between the two groups both before and after the intervention (p 0.001). From the beginning of the intervention until one month later, there was a significant increase (*p* = 0.175). In the same context, Bai & Manomozhi [24], reported that the majority of virtual reality students were satisfied post implementation of the training program and [33] who illustrated that nursing student’s post VR simulation program received higher scores of satisfaction than control group. Furthermore; Chang et al. [34] who clarified that Virtual reality simulation increases nursing student’s satisfaction post application of the training program. In the researcher point of view, this result may be due to the midwifery nursing student uses the virtual reality glasses individually, which increases his self-confidence and satisfaction.

Meanwhile the result wasn’t in consistent with [35] that cleared that there was no significant difference in satisfaction between VR and traditional nursing skills practice groups. From the perspective of the researcher, Using VR may result in cyber sickness; including nausea, dizziness, and headache these lead to not satisfaction. Thus, future research should concentrate on the detrimental impacts of VR, such as impaired vision and confusion. These outcomes demonstrated the critical function of virtual reality-assisted learning on enhancing performance levels regarding psychomotor competence in midwifery nursing students, Of course, with all these results that have been reached regarding the satisfaction of participants in various researches with the use of virtual reality technology as a new method of practical training, especially among nursing students, it can be emphasized that it should be used and applied in all practical fields to improve performance at all levels.

However, several recent studies suggest that learning gains are not solely due to the immersive nature of VR, but rather to the instructional design elements it enables—particularly safe repetition, experimentation, and immediate feedback [36, 37]. VR provides a psychologically safe space where students can practice complex procedures like Leopold’s maneuver repeatedly without fear of harming patients. It also allows for real-time corrective feedback, which is critical in developing clinical proficiency. Therefore, while VRs immersive environment may enhance motivation and interest, the sustained learning outcomes are more likely linked to how it supports active learning principles, such as deliberate practice, feedback loops, and reflective action. This is particularly relevant in midwifery education, where skill acquisition and confidence are built through repeated hands-on experience.

## Limitation

### This study has several limitations that should be acknowledged

- The short duration of follow-up, limited to only one week after the intervention, does not allow for the evaluation of long-term retention of clinical skills. Future studies with extended follow-up periods are needed to assess the sustainability of skill acquisition over time.
- Despite that, some result had high success rates such as (97%); we cannot generalize the result because the sample size is small. coupled with the fact that student participation was sought from one university only, impacts negatively on the generalizability and transferability of findings. VR equipment constraints as motion cyber sickness during training that had some effect on learning outcomes.
- The study may be subject to assessment bias, as the assessors were not blinded to group allocation. This lack of blinding could have inadvertently influenced the evaluation of student performance, particularly in subjective skill assessments.
- The researchers were required to undergo extensive training on the technical operation of the virtual reality modules to administer the intervention, which made it impossible for them to be blinded to the treatment allocation
- Prior experience with VR was assessed by self-report, which exposes midwifery nursing students to recall bias and may have an effect on accuracy of their past exposure to VR technology.
- Similar limitations have been noted in previous VR-based studies in nursing and medical education, which also emphasized the need for larger samples, multi-center designs, and long-term evaluation to draw more definitive conclusions [36, 37]
- Despite these limitations, the study provides valuable insights into the potential of VR in midwifery education, and it serves as a foundation for future large-scale, multi-center trials with more robust methodological designs

## Conclusion

The study concluded that midwifery nursing students who received virtual reality-assisted training demonstrated better psychomotor competence, superior performance, and greater satisfaction compared to those trained using traditional simulation methods. However, although the VR group showed higher knowledge scores, the difference was not statistically significant.

### Recommendations

#### Based on the findings of this study, it was recommended that:-

1. Integrate Virtual Reality-Assisted Learning (VRAL) as a Complementary tool to traditional manikin-based simulation. While VR offers immersive, scenario-based decision-making practice, manikin-based training remains essential for developing tactile and hands-on physical examination skills. A blended approach allows students to benefit from both methods and accommodates various learning styles.
2. Define Optimal Frequency of VR Sessions: It is recommended that students engage in 3 to 5 structured VR sessions throughout the clinical training period. These sessions should be spaced out (once per week) to allow for reinforcement, reflection, and retention of psychomotor and clinical reasoning skills.
3. Implement Structured Feedback After Each VR Session: Each VR session should be immediately followed by guided debriefing and formative feedback, delivered by trained instructors. This feedback loop is essential to consolidate learning, correct misconceptions, and build student confidence.
4. Provide Targeted Faculty Training: Instructors and clinical educators should undergo a 2–3 day hands-on training workshop prior to VR implementation. This training should cover device operation, curriculum integration strategies, troubleshooting common issues, and interpreting VR performance metrics. Training could be delivered in collaboration with VR developers or academic simulation centers.
5. Pilot VR Integration before Full-Scale Adoption: Conduct a pilot implementation phase within the institution to identify logistical challenges, technical barriers, and student perceptions. This step ensures smoother integration and contextual adaptation based on institutional resources.
6. Align VR Use with Learning Objectives and Clinical Outcomes: Ensure that the use of VR is driven by clear educational goals, mapped to course competencies and national clinical standards. VR activities should not be used merely for engagement, but to enhance measurable skill acquisition and decision-making capacity.
7. Encourage further large-scale research to evaluate the long-term impact of VRAL on clinical performance and patient outcomes in real-world healthcare settings

## Data Availability

The data that support the findings of this study are available from the corresponding author upon reasonable request.
